#  A Novel Approach to the Development of Anticarcinogenic Vaccines 

**Published:** 2010

**Authors:** A.N. Glushkov, S.V. Apalko, M.L. Filipenko, V.A. Matveeva, A.Yu. Bakulina, V.G. Lunin, M.V. Kostyanko

**Affiliations:** Institute of Human Ecology, Siberian Branch, Russian Academy of Sciences; Institute of Chemical Biology and Fundamental Medicine, Siberian Branch, Russian Academy of Sciences; Gamaleya Research Institute of Epidemiology and Microbiology; Kemerovo State University

## Abstract

Human exposure to chemical carcinogens is an important etiological factor in cancer diseases. In this article, we will discuss a new approach to the development of anticarcinogenic vaccines. The main task in our research was to select a benzo[a]pyrene immunomimetic peptide considered as a hapten-specific component. For this purpose, we synthesized carcinogen-protein conjugates and prepared mono- and polyclonal antibodies to benzo[a]pyrene. Phage display technology was used to select the benzo[a]pyrene immunomimetic peptide, followed by an evaluation of the immunological properties of the obtained peptide. The obtained benzo[a]pyrene immunomimetic peptide could only simulate chemical carcinogens in the frame of the pIII protein. As a result, we prepared a recombinant protein composed of the benzo[a]pyrene immunomimetic peptide and pIII-encoding sequences. Using ELISA, we demonstrated that the recombinant protein specifically interacts with the anti-benzo[a]pyrene monoclonal antibody (mAB B2). Using molecular modeling, we predicted the 3-D structure of the mAB B2 active center and analyzed the characteristics of its interaction with different polycyclic aromatic hydrocarbons, as well as with the benzo[a]pyrene immunomimetic peptide. Thus, a comprehensive analysis of the results of the obtainment of hapten-specific components of anticarcinogenic vaccines allowed us to outline a strategy for future development in this direction.

##  INTRODUCTION 

 The UN Health Agency has reported that more than 8 million people die from cancer every year. This reinforces the need to develop a novel therapeutic strategy based on antitumor vaccines. Unfortunately, such vaccines commonly target the existing disease rather than its cause. 

 Data from the World Health Organization (WHO) indicate that 90% of cancer cases are a result of the action of environmental carcinogenic agents. The bulk (70–80%) of such agents is made up of chemicals, including widely circulating polycyclic aromatic hydrocarbons (PAHs). It is an easy guess to assume that the identification of carcinogenic substances and their elimination from the sphere of human activity could serve as effective cancer prophylactic. However, such an approach is virtually impossible to pursue because of many factors. Therefore, the creation of antitumor defense by anticarcinogenic vaccines buttressing the immunological barrier in animals (including humans) against carcinogenic chemicals, seems necessary. 

Chemical carcinogens are low-molecular substances that cannot, in themselves, induce an immune response. In 1937, Creech and Franks first synthesized conjugates of carcinogens with high-molecular carriers - blood serum proteins. They found that immunization of these conjugates leads to synthesis of specific anticarcinogenic antibodies (ABs). At the same time, some inhibition of carcinogen-induced tumor progression was noted following pre-immunization, and the idea that the approach could be applied to prevent tumor development in humans was first put forward [1].

In 1981, Moolten and associates took the next step in the development of anticarcinogenic vaccines. They prepared protein conjugates with a structural analog of carcinogen, which in itself cannot induce a tumor. The pre-immunization of animals with this conjugate essentially decreased the probability of tumorigenesis caused by an actual carcinogen [2].

A novel approach was used by Chagnaud and associates. In 1992, they reported the preparation of an anti-idiotypic monoclonal antibody (mAB) against benzo[a]pyrene (BP). The second anti-idiotypic mAB carries the internal image of a carcinogen and can induce the synthesis of the first ABs against the carcinogen without the use of carcinogen-protein conjugates. The inhibitory effect of the second mABs on the development of chemically induced tumors was described in 1993 [3].

In the mid-1980s and afterward, Silbart and associates focused their efforts on the induction of specific secretory ABs in the gastrointestinal and respiratory mucosa by combining carcinogen-protein conjugates with various adjuvants to create a barrier preventing carcinogen transport between the environment and the body. In a review published in 1997, Silbart directly raised the issue of the future use of anticarcinogenic vaccines in humans [4].

 Since conjugates of carcinogens or their analogs with carrier proteins can lead to iatrogenic induction of tumors, and the introduction of anti-idiotypic ABs – to allergic and autoimmune diseases, the proposed approaches are inapplicable in the development of anticarcinogenic vaccines for animals, including humans. 

 We offer a fundamentally novel approach in anticarcinogenic vaccine development implying the use of a peptide as a hapten-specific component that can induce specific anticarcinogenic ABs. Since BP is one of the most active and widely distributed PAHs and an absolute carcinogen for humans, we set out to prepare a peptide immunomimetic of BP. 

##  MATERIALS AND METHODS 

**Synthesis of conjugates**

*PAH-protein conjugates* of BP, benz[a]anthracene, anthracene, chrysene, and pyrene (Aldrich, Germany) were synthesized by means of covalent binding of haptene aldehyde groups with the amino groups of the carrier protein (bovine serum albumin [BSA] or hexokinase) [5].

*Peptide-cBSA conjugates* : 2 mg of the synthetic peptide and 10 mg of 1-ethyl-3-(3-dimethylaminopropyl)carbodiimide hydrochloride (EDC) were added to 700 µL of solution containing 2 mg of cationized BSA (cBSA) [6], incubated for 2 hours, and dialyzed six times against 1 L of H _2_ O.

**Immunization of laboratory animals**

*Preparation of hybridomas producing mABs specific to BP* : hybridomas were prepared by the fusion of murine Sp2/0 myeloma cells and female Balb/c mouse splenocytes following immunization with the BP-BSA conjugate [7], as described by Kohler G. and Milstein C. [8].

*Preparation of polyclonal ABs against BP* : rabbits were immunized with 2 mg of the BP-BSA conjugate by weekly intramuscular injections for three weeks. The first injection was performed with the mixture of the antigen with a complete Freund’s adjuvant (CFA) (Sigma, USA); the second – with the mixture of the antigen with a incomplete Freund’s adjuvant; and the third – with the antigen in PBS. Then, supporting injections were introduced once in two weeks. Blood was taken two months after the beginning of immunization – every other week.

*Immunization of animals with a chimeric protein containing a BP immunomimetic peptide* : Balb/c mice were immunized by intraperitoneal injections of a chimeric protein four times once every two weeks. For the first injection, the antigen was mixed with CFA. Other injections were performed using the antigen mixture with an incomplete Freund’s adjuvant. The amount of the antigen was 100–150 mg. The blood serum was tested for specific ABs against PAH, beginning from the first injection.

*Purifucation of anti-BP ABs* was carried out by affinity chromatography on columns filled with PAH-hexokinase-Sepharose 4B [9]. The replacement of the carrier protein enabled to avoid a preliminary purification of the antiserum from admixing ABs against the protein carrier used for immunization (anti-BSA ABs).

**ELISA**

*Identification of specific anti-PAH-BSA ABs by ELISA* : PAH-BSA conjugate (5 µg/mL, 100 µL in each well) was sorbed into wells of a polystyrene 96-well plates (Medpolymer, Russia) for 12 hours at 4°C. Nonspecific binding sites were blocked with 0.5% BSA in PBS, pH 7.2–7.4, containing 8 g of NaCl, 0.2 g of KCl, 2.68 g of Na _2_ HPO _4_ × 7H _2_ O, 0.24 g of KH _2_ PO in 1 L of water, then100 µL of blood serum samples serial dilutions in PBS containing 0.05% Tween-20 (PBST) and 0.5% BSA were added into the wells. Unbound material was removed by washing with PBST and PBS, and bound ABs were detected by treatment with an anti-mouse IgG horseradish peroxidase (Biosan, Russia) conjugate, followed by staining with TMB (Fluka, Switzerland). The optical density was determined on a microplate reader (FFM, Russia) at 450 nm.

*To detect a specific binding of the chimeric protein with anti-BP ABs* , The mono- or polyclonal AB to BP (5 µg/mL) was sorbed into wells of a polystyrene 96-well plate. Following blocking, serial dilutions of the chimeric protein in PBST containing 0.5% BSA (100 µL per a well) were added and incubated. The plates were then thoroughly washed, and 100 µL of rabbit serum against a cellulose-binding domain (CBD) was added into each well followed by incubation, while the bound recombinant proteins were detected with an anti-rabbit IgG horseradish peroxidase conjugate as described above. The experiment was triplicated for estimation of reproducibility.

*Competitive ELISA* : conjugate BP-BSA (5 µg/mL) was sorbed into wells of a polystyrene 96-well plate. Following blocking, a mixture of mAB B2 of equal concentration with varied amounts of a competitor (PAH or synthetic peptide) was added into the wells. The mAB B2-competitor mixtures (total volume 100 µL, each) were preincubated for 30 min at 37°C and shaken gently. All tested samples were diluted with PBST containing BSA. The plates were incubated for 1 hour at 37°C and shaken gently. Then, following thorough washing with PBST, the bound mABs were detected with an anti-mouse IgG horseradish peroxidase conjugate as described above. The experiment was triplicated for estimation of reproducibility.

*Affinity selection of phage display peptide library* was carried out according to the recommended protocol to the Ph.D-12 ^TM^ kit (New England BioLabs), with additional modifications [10].

*Chemical synthesis of peptides* by the method of activated esters in solution was performed in the Laboratory of Organic Synthesis, Institute of Chemical Biology and Fundamental Medicine, SB, RAS.

**Molecular modeling**

 . 

Optimum patterns for the AB structure modeling by homology were matched using the BLAST server. Modeling was performed using the Modeller9v1 software. Molecular docking was performed using AutoDock version 4.0. Construction of the model for a peptide comprising the pIII protein was performed using the Rosetta program for *de novo* modeling [11].

##  RESULTS AND DISCUSSION 

**Synthesis of PAH-protein conjugates for preparation and analysis of antibodies**

 A major shortcoming of known methods of PAH-protein conjugate synthesis including the preparation of BP conjugates with proteins is the formation of a polymer, which considerably decreases the yield of the soluble fraction and, as a result, makes this conjugate unsuitable for immunoassay. So, the main task in this set of studies was to prepare well-soluble hapten-protein conjugates that contain a minimum of the polymeric admixture and are stable without the need for specific stabilizers. 

We applied a method of covalent hapten-protein binding via the formation of an azomethine bond between an aldehyde group of hapten and amine groups of protein [5]. The use of BP aldehyde for the synthesis of conjugates with proteins enabled good results: animal blood sera with high titers of anti-BP ABs were obtained. Immunization with a hapten bound with one carrier protein (for example BSA) followed by the detection of ABs against this hapten based on another carrier protein (hexokinase) was found to be highly effective in both the analysis of anti-BP ABs in direct and competitive ELISA and the one-step preparation of affinity-purified ABs against PAH [9].

**Preparation and immunochemical characterization of a monoclonal antibody against benzo[a]pyrene**

There are several anti-BP mABs available in the world currently (USA, Czech Republic, Japan). They were raised mainly for the development of ELIZA-based test-systems to detect PAH pollutants in the environment, as well as their metabolites and DNA-adducts in the biological fluids of animals, including humans [12–14]. The main shortcoming of these mABs – in the case of hapten-specific vaccine component preparation – is the insufficient specificity of their binding with BP, compared with noncarcinogenic PAH. Besides, the capability of the abovementioned mABs to bind with hydrophobic endobiotics (steroid hormones) and aromatic aminoacids hasn’t been studied yet . So, the key stage in our work was the preparation of a highly specific anti-BP mAB and the analysis of its cross-reactions with other PAHs, steroid hormones, and aromatic aminoacids.

Among the obtained murine hybridoma clones, the clone B2 was chosen producing IgG mAB, which had no affinity to the anthracene-BSA conjugate and had low affinity to chrysene-BSA and pyrene-BSA conjugates. The mAB B2 most effectively binds BP and benz[a]anthracene, a putative human carcinogen [7].

 We checked for the possibility of cross-reaction of the mAB B2 with aminoacids, such as tryptophan and phenylalanine, based on the belief that the presence of an aromatic ring is one of the requirements of the interaction of anti-PAH AB with other substances. It is known that one aryl hydrocarbon receptor is implicated in signal transduction from PAH and endogenous substrates (in particular, estrogens); so, we also studied the cross-reaction of the mAB B2 with these substances and found no binding. This excludes the probability of preparing an anticarcinogenic vaccine with a side effect such as inducing autoimmune reactions against endogenous ligands. 

**Preparation and characterization of a benzo[a]pyrene immunomimetic peptide**

We applied the phage display technique in the search for a BP immunomimetic peptide. The procedure of affinity selection included incubation of the Ph.D-12 ^TM^ initial library with mono- and polyclonal ABs to BP and washing from AB-unbound and elution of bound bacteriophages. The preliminary procedure of bacteriophage exhaustion with intact murine or rabbit IgG was substituted with cross-mapping of ABs in the third round of selection; i.e., the first two rounds were carried out on single species ABs, for instance mAb B2, and polyclonal ABs against BP were mapped with the obtained bacteriophage population in the third round, and vice versa. The proposed approach should favor the selection of high-affinity bacteriophage clones.

Five resulting bacteriophage clones were produced that specifically interact with mAB B2. Four clones resulted from cross-selection, when the two first rounds were carried out on mAB B2, and the last round – on polyclonal ABs to BP. One clone was produced when all selection rounds were carried out on mAB B2. DNA sequencing of these clones, followed by translation, demonstrated that all five clones had an identical aminoacid sequence of the recombinant peptide: LeuHisLeuProHisHisAspGlyValGlyTrpGly [10, 15].

**Fig. 1:**
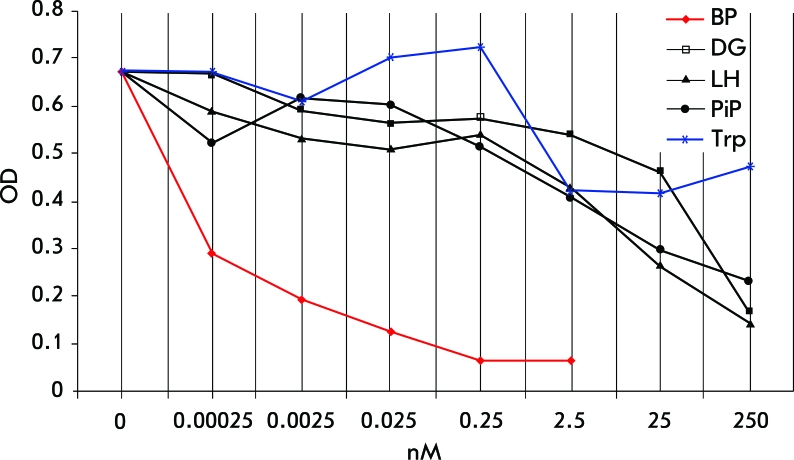
Competitive inhibition of mAb B2 binding with immobilized conjugate BP-BSA by BP, DG, LH, PiP, and Trp.

 The BP immunomimetic peptide (named PiP) was synthesized for further study of its immunochemical properties. Since two halves of the PiP, LeuHisLeuProHisHis (LH-peptide) and AspGlyValGlyTrpGly (DG-peptide), were synthesized first and then linked, we also evaluated LH- and DG-peptides for specific interaction with mAB B2. 

 Since the PAH structural mimicry is supposed to depend on the presence of a tryptophan residue within the peptide sequence, tryptophan was used as a negative control. 

Synthetic peptides were found to compete with the BP-BSA conjugate for the binding with mAB B2. However, their binding force is substantially weaker than that of BP. Tryptophan (Trp) did not demonstrate any significant competition for binding with mAB B2 (Fig. 1). This fact suggests that Trp can specifically bind with ABs against the PAH group of chemical carcinogens, only when in context with other amino acid residues of these peptides.

 The nature of LH-peptide binding with mAB B2 remains an enigma. One can speculate that a very complex interaction takes places between the PiP peptide and mAB B2, which cannot be explained by the fact that Trp or other hydrophobic residues structurally mimic BP. 

Analysis of blood sera from mice immunized with peptide–cBSA conjugates revealed the presence of ABs to benz[a]anthracene and anthracene. However, their level is an order of magnitude lower than that of anti-PAH ABs induced by the immunization of mice with BP-BSA [11].

Several reports on the production of the peptide mimetic of low-molecular compounds have shown that the initial conformation of peptides present on the surface of a bacteriophage carrier can change when the peptide is disengaged, or undergo additional modification. These alterations are crucial for ABs to recognize a peptide [16].

Thus, using the Rosetta software, we constructed a model for the peptide portion within the bacteriophage M13 pIII protein. The model assumes that the Trp side radical is localized on the surface of the protein [11]. It is likely that the structure of a peptide immunomimetic enabling mimicry of PAH-type carcinogens is possible only within the context of the pIII protein. In this context, our efforts therefore focused on the production of a recombinant protein composed of the BP immunomimetic peptide and bacteriophage pIII protein.

**Preparation and characterization of a recombinant protein containing the benzo[a]pyrene immunomimetic peptide**

Several approaches in gene engineering are known to enable an increase in the expression level and stability of transgenic proteins in a bacterial system, the facilitation of the testing procedure, and to enhance efficiency in protein purification. One of these approaches is a fusion technique directed toward the synthesis of chimeric proteins. It is based on the linkage of two genes (a gene for the antigene component and that encoding a carrier protein) in one reading frame, which leads to the synthesis of a chimeric protein in the bacterial system [17].

Using this technology, we produced and characterized the chimeric protein whose antigen component comprises amino acid sequences of a BP immunomimetic peptide and the pIII protein of bacteriophage M13, on the basis of which the Ph.D-12 ^TM^ library was constructed. A CBD-domain of *Anaerocellum thermophilum* endoglucanase was used as a carrier. It can interact with high affinity with a cellulose sorbent, thus enabling the isolation and purification of the recombinant protein containing the immunomimetic peptide.

**Fig. 2:**
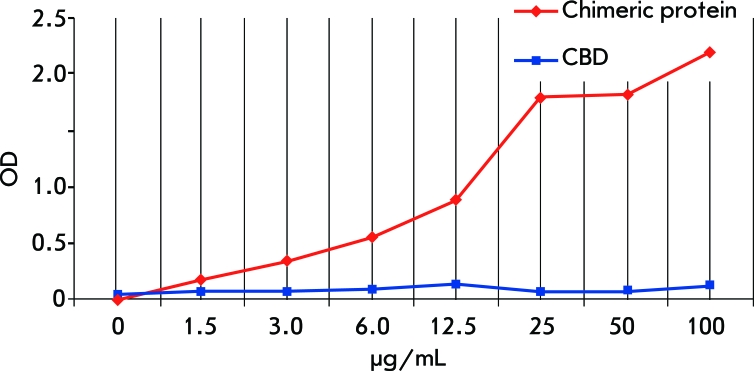
Binding of chimeric protein containing benzo[a]pyrene immunomimetic peptide with mAB B2.

Noncompetitive ELISA was used in the study of the interaction between mAB B2 and the produced chimeric protein containing the antigen component (BP immunomimetic peptide within the pIII protein) and the carrier protein (CBD). CBD was used as the negative control. The ability of the chimeric protein containing the immunomimetic peptide to bind with mAB B2 sorbed on a plastic was found to be dose-dependent (Fig. 2). The chimeric protein did not bind polyclonal murine and rabbit ABs to BP.

Balb/c mice were intraperitoneally immunized with the chimeric protein to test for the ability of the immunomimetic peptide within the bacteriophage pIII to induce ABs against PAH. A low level of anti-PAH ABs was detected in the blood serum of immunized mice. The most prominent binding was detected between the ABs and anthracene. At the same time, the blood serum of mice immunized with the bacteriophage recombinant clone containing the BP immunomimetic peptide within the pIII protein contains a mAB against BP, wherein the titers are comparable with those in the positive control – immunization with the BP-BSA conjugate [10, 15].

 With the aim to find approaches to enhancing chimeric protein immunogenicity in relation to BP, we studied – using molecular modeling – the spatial structure of the mAB B2 active center and the features characterizing its interaction with PAHs and the immunomimetic peptide. 

**Peculiarities of the interaction between mAB B2 and benzo[a]pyrene immunomimetic peptide**

A model for the mAB B2 Fab-fragment was created by the method of homology using the determined primary structures of heavy and light chains. The average binding energy between the mAB B2 Fab-fragment and several PAHs calculated using molecular docking software correlated with the experimental data on cross-reactivity between mAB B2 and these PAHs, thus confirming the validity of the created model [11].

Two pockets for PAH binding possibly exist in the active center of mAB B2, as was determined by molecular docking. The best position for BP and other PAH binding was determined to be between the third loops of the light and heavy chains of mAB B2 (this pocket was named P1). Two variants of BP docking in the P1 pocket were determined: the vertical and the horizontal, the first and the second one, respectively. The second pocket between the second loop of the light chain and the third loop of the heavy chain was less profound and less preferable for PAH binding, judging from the higher binding energy predicted by the docking (this pocket was conditionally named P2) [11].

**Fig. 3:**
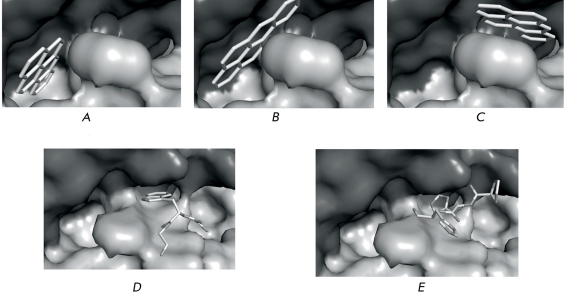
The structure models of BP, GlyTrpGly, and ProHisHis complexes with the active center of the mAB B2 Fab-fragment. BP binding in positions P1P1 ( *A* ), P1P2 ( *B* ), and P2 ( *C* ); GlyTrpGly ( *D* ) and ProHisHis ( *E* ) binding in position P2.

A number of molecular docking calculations for the mAB B2 Fab-fragment with tripeptides comprising PiP have been carried out with the aim of modeling the interaction between the LeuHisLeuProHisHisAspGlyValGlyTrpGly peptide and mAB B2. None of the tripeptides binds with ABs in the region of the first pocket. Several tripeptides (HisLeuPro, LeuProHis, ProHisHis, and GlyTrpGly) have bound with Ab in the region of the second pocket (Fig. 3). Three tripeptides are convergent in the presence of a histidine residue, which is not shielded by other amino acid residues. This explains the LH-peptide’s ability to compete with the BP-BSA conjugate for mAB B2.

 At the same time, tryptophan, being within the BP immunomimetic peptide, obviously plays the key role in the binding of mAB B2, if the latter is exposed to the protein’s surface. This immunomimetic peptide structure is possible within the pIII protein structure. 

 When the presence of the second binding pocket in the active center of mAB B2 is taken into account, one can explain the fact that the chimeric protein containing the immunomimetic peptide actively binds only to mAb B2, but not other polyclonal ABs against BP. 

 It is likely that in the process of recombinant bacteriophage selection on mAb B2, the peptides were selected by their binding with the second pocket as the most desired one. It is possible that the initial library contained no peptide capable of specifically binding with the deeper first pocket. The fact that no clones capable of specifically binding with the mAB B2 were found among the recombinant bacteriophages that resulted from the affinity selection on polyclonal ABs, as well as the fact that all five clones had an identical amino acid sequence, confirms the abovementioned hypothesis. 

The experimentally revealed weak reverse-mimicry * in vivo* , i.e. weak immune reaction between AB and PAH, when mice were immunized with chimeric protein can be explained as follows.

 As mentioned above, tryptophan has the closest structural similarity to BP among all side radicals. Superposition of the tryptophan and BP structures at the first position of the P1 pocket demonstrates that tryptophan, being within the polypeptide chain, cannot bind to such a deep cavity, because the length of BP exceeds that of the tryptophan side radical. At the same time, BP structural mimicry by tryptophan, in combination with some other side aminoacid radicals, is possible in the second position of the P1 pocket. It is likely that some part of AB induced by immunization with the immunomimetic peptide has a cavity for binding which is similar to the P2 pocket: therefore, tryptophan binding to AB does not generally bind to BP. Other parts of AB can possess a cavity for the binding of the P1 pocket’s second position, enabling them to bind PAHs, including BP. 

##  Conclusion 

 The use of conjugates of chemical carcinogens (or their structural analogs) with macromolecular carriers as vaccines is completely unacceptable in the immune prophylaxis of malignant tumors in humans because of the risk that the vaccine itself can induce a tumor. The hybridoma technique for the production of anti-idiotypic ABs has limitations in the optimization of the immunogenic properties of target vaccines. In addition, it is complex and expensive. 

 The proposed approach, i.e. the production of immune peptides that mimic chemical carcinogens using phage display technology, is preferable. 

 The mAB B2 obtained in our experiments possesses high specificity to BP, low cross-reactivity with noncarcinogenic PAHs, and does not react with endobiotics. Moreover, molecular docking showed that the predicted average energy for dibenzo[a]pyrene binding to the mAB B2 Fab-fragment is –8.91 kcal/mol, which is higher than the values for BP and other PAHs. We have established a direct correlation between the predicted binding energy and the experimentally measured PAH cross-reactivity. Based on this, one can surmise that the usage of mAB B2 in phage display technology could be effective in the search for immune peptides that mimic not only BP, but other PAHs with higher carcinogenic activity as well. 

It is important to note that anti-idiotypic mABs are produced when animals are immunized with polyclonal ABs to BP [3]. In this context, the right strategy going forward will be to use new molecular targets in the search for PAH immunomimetic peptides. In our opinion, the use of the recombinant Fab-fragment of the mAB B2 with its second pocket removed via site-directed mutagenesis seems to be the most successful avenue. The recombinant bacteriophages resulting from the selection on such AB must be tested for binding with polyclonal ABs against BP.

 The second method in enhancing the immunogenicity of target vaccines against carcinogenic PAHs would be the use of other phage libraries and/or optimization of the recombinant peptide structure via point mutations. This approach could enable the production of a peptide inducing a highly specific immune response to BP and more carcinogenic PAHs. 

## Abbreviations

CBD: cellulose-binding domain
OD: optical density
BP: benzo[a]pyrene
BSA: bovine serum albumin
AB: antibody
mAB: monoclonal antibody
ELISA: enzyme-linked immunosorbent assay
PAH: polycyclic aromatic hydrocarbon
